# Incidental Finding of Lipomatous Hypertrophy of the Right Atrial Free Wall in an Elderly Female With Severe Pulmonary Hypertension: Early Detection by Multimodality Imaging

**DOI:** 10.7759/cureus.50665

**Published:** 2023-12-17

**Authors:** Andrea Sonaglioni, Gian Luigi Nicolosi, Gaetana Anna Rispoli, Michele Lombardo

**Affiliations:** 1 Cardiology, Istituto di Ricovero e Cura a Carattere Scientifico (IRCCS) MultiMedica, Milan, ITA; 2 Cardiology, Policlinico San Giorgio, Pordenone, ITA; 3 Radiology, Istituto di Ricovero e Cura a Carattere Scientifico (IRCCS) MultiMedica, Milan, ITA

**Keywords:** emergency department, multi-instrumental evaluation, incidental cardiac finding, lipomatous atrial hypertrophy, right atrial pseudomass

## Abstract

Lipomatous atrial hypertrophy (LAH) is a benign cardiac lesion characterized by fat accumulation in the interatrial septum that spares the fossa ovalis. It is associated with obesity and is more frequently observed in elderly and female patients. It is most often detected as an incidental finding on transthoracic echocardiography (TTE). The deposition of adipose tissue may rarely involve both the interatrial septum and the right atrial (RA) free wall. Herein, we describe an extremely rare case of LAH limited to a portion of the RA free wall only, mimicking a myxoma or a thrombotic formation. A multi-instrumental evaluation comprehensive of TTE implemented with pulsed-wave tissue Doppler imaging (PW-TDI), transesophageal echocardiography (TEE), and computed tomography (CT) angiography, performed during the patient's stay in the emergency department, allowed to quickly diagnose the benign RA pseudomass.

## Introduction

Lipomatous atrial hypertrophy (LAH) is a relatively uncommon benign entity characterized by excessive fat deposition within the atrial septum. It can be found incidentally on transthoracic echocardiography (TTE) [1]. In rare cases, the deposition of adipose tissue may involve both the interatrial septum and the right atrial (RA) free wall. We present an unusual case of LAH limited to a portion of the RA free wall only, early diagnosed by a multi-instrumental evaluation comprehensive of TTE implemented with pulsed-wave (PW) tissue Doppler imaging (TDI), transesophageal echocardiography (TEE), and computed tomography (CT) angiography.

## Case presentation

A 78-year-old female (body surface area (BSA) 1.60 m^2^, body mass index (BMI) 20.2 kg/m^2^), affected by arterial hypertension and dyslipidemia, was admitted to the emergency department of our hospital due to exertional dyspnea lasting two weeks. She had a previous history of cerebellar stroke 11 years before and splenic injury due to motor vehicle accident treated with splenectomy nine years before, followed by the occurrence of JAK2 V617F-positive essential thrombocythemia (ET). She was chronically treated with acetylsalicylic acid 100 mg/day, amlodipine 5 mg/day, atorvastatin 40 mg/day, and oncocarbide 4.5 g/week.

At hospital admission, blood pressure was 110/70 mmHg, heart rate was 90 bpm, and arterial oxygen saturation was 74%. The initial laboratory workup is shown in Table 1. Arterial blood gas analysis revealed respiratory alkalosis with hypoxemia and hypocapnia, whereas mild iron deficiency anemia was diagnosed by blood tests.

**Table 1:** Initial laboratory workup on hospital admission. Hb, hemoglobin; HCT, hematocrit; NT-proBNP, N-terminal pro b-type natriuretic peptide; PaO_2_, arterial partial pressure of oxygen; PaCO_2_, arterial partial pressure of carbon dioxide; PLTs, platelets

Test	Results	Reference range
pH	7.49	7.35–7.45
PaO_2_ (mmHg)	37.0	80.0–100.0
PaCO_2_ (mmHg)	29.6	32.0–45.0
White blood cell count (x 10^3^/μl)	3.97	4.00–11.00
Hb (g/dl)	10.4	12.5–16.0
Red blood cell count (x 10^6^/μl)	3.55	4.00–5.00
HCT (%)	32.5	34.0–48.0
PLTs (x 10^3^/μl)	272	150–400
Creatinine (mg/dl)	0.65	0.50–0.80
D-dimer (ng/ml)	958	0–682
C-reactive protein (mg/dl)	0.5	0.00–0.50
NT-proBNP (pg/ml)	453	0–450
Troponin I (ng/ml)	0.00	0.00–0.04

Chest X-ray showed cardiomegaly with bilateral hilar and parenchymal congestion and concomitant bilateral pleural effusion. A sinus rhythm with incomplete right bundle branch block and secondary repolarization abnormalities in V1-V3 leads was detected on electrocardiogram.

A bedside TTE highlighted a moderate dilatation of right heart chambers cavity sizes with a normal right ventricular systolic function (tricuspid annular plane systolic excursion = 25 mm) and severe tricuspid regurgitation and severe pulmonary hypertension (PH), as assessed by tricuspid regurgitation velocity (TRV) magnitude (TRV = 3.7 m/sec); on the other hand, left-sided cardiac chambers were small-sized, and the left ventricular systolic function was supernormal (estimated Simpson's biplane ejection fraction = 69%), in absence of a mitral and/or aortic valvular disease.

**Figure 1:**
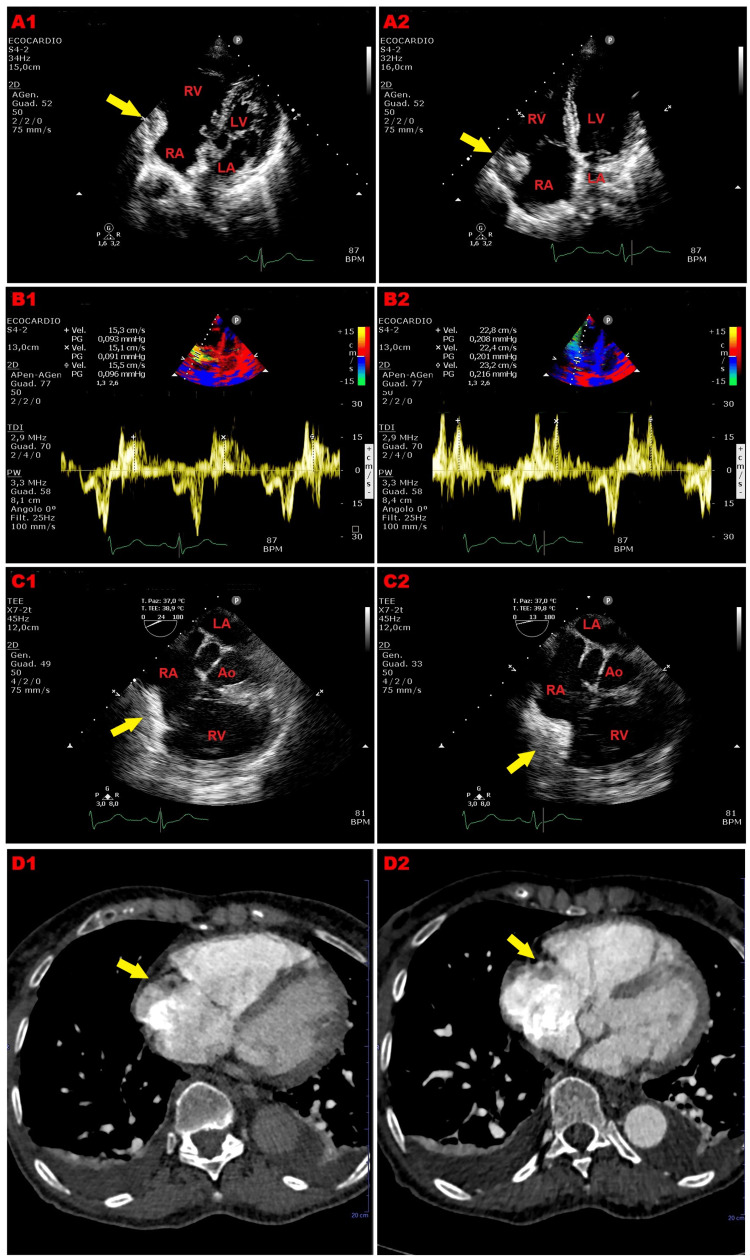
Multi-instrumental evaluation of the suspected right atrial mass. Panel A1: Transthoracic echocardiography. Apical four-chamber view focused on the right-sided chambers (end-diastolic frame), showing moderate dilatation of right heart chambers cavity sizes and significant hypertrophy and hyperechogenicity of the anterior right atrial free wall in proximity of the antero-lateral edge of the tricuspid annulus (yellow arrow). Panel A2: Transthoracic echocardiography. Apical four-chamber view focused on the right-sided chambers (systolic frame), revealing a suspected right atrial mass, occupying the infero-lateral portion of right atrial cavity (Figure 1, Panel A2, yellow arrow). Panel B1: Peak antegrade velocities of the suspected right atrial mass, obtained by placing a 5-mm sample volume of PW-TDI at the level of the mobile portion of the same mass, at end-diastole. Panel B2: Peak antegrade velocities of the suspected right atrial mass, obtained by placing a 5-mm sample volume of PW-TDI at the level of the mobile portion of the same mass, in mid-systole. Panel C1: Mid-esophageal view 24° focused on the right-sided chambers (end-diastolic frame), showing lipomatous hypertrophy involving the anterior right atrial free wall portion near the antero-lateral edge of tricuspid annulus (yellow arrow). Panel C2: Mid-esophageal view 13° focused on the right-sided chambers (mid-systolic frame), revealing infolding of the lipomatous right atrial free wall toward the right atrial cavity (yellow arrow). Panel D1: Contrast-enhanced axial CT scan, confirming both the enlargement of the right heart chambers and the lipomatous hypertophy of the anterior right atrial free wall, which was homogenously hypodense (yellow arrow). Panel D2: Contrast-enhanced axial CT scan, showing the systolic infolding of the lipomatous anterior right atrial free wall toward the cavity (yellow arrow). Ao, aorta; CT, computed tomography; LA, left atrium; LV, left ventricle; PW, pulsed wave; RA, right atrium; RV, right ventricle; TDI, tissue Doppler imaging

A careful analysis of right-sided cardiac chambers at end-diastole revealed a significant hypertrophy and hyperechogenicity of the anterior RA free wall in proximity of the antero-lateral edge of the tricuspid annulus (Fig. 1, Panel A1, yellow arrow).

During the systolic phase of the cardiac cycle, the infero-lateral portion of the RA cavity resulted to be filled by a peduncolated intra-atrial mass, mimicking a myxoma or a thrombotic formation (Fig. 1, Panel A2, yellow arrow). By placing a 5-mm sample volume at the level of the free mobile portion of the suspected RA mass, PW-TDI allowed to obtain a lower peak antegrade velocity (Va) at the end-diastole (average peak Va 15.3 cm/s) (Fig. 1, Panel B1) and a higher Va in the mid-systole (average peak Va 22.8 cm/s) (Fig. 1, Panel B2). The velocity and direction of the movements of the suspected RA mass were concordant with surrounding myocardial and tricuspid valve tissues, in synchrony with cardiac cycle. Accordingly, myxoma and thrombotic formation were excluded and a lipomatous hypertrophy of the anterior RA free wall with systolic infolding into the RA cavity was diagnosed.

TEE performed in the ED confirmed lipomatous hypertrophy involving the anterior RA free wall near the antero-lateral edge of the tricuspid annulus (diastolic RA free wall thickness = 12 mm) (Fig. 1, Panel C1, yellow arrow) with systolic infolding toward the RA cavity (Fig. 1, Panel C2, yellow arrow).

An urgent computed tomography (CT) angiography excluded pulmonary embolism and confirmed enlargement of the right heart chambers with concomitant congestive signs; the anterior RA free wall was hypertrophic and homogenously hypodense (Hounsfield units = − 60) (Fig. 1, Panel D1, yellow arrow), as for the fatty composition, and showed a systolic motion toward the RA cavity (Fig. 1, Panel D2, yellow arrow).

Considering the clinical and instrumental finding of the right-sided heart failure due to severe PH likely secondary to ET, the patient was immediately transferred to the Pneumology Division. During the hospitalization, she was treated with intravenous diuretics (furosemide 40 mg/day and potassium canrenoate 100 mg/day), enoxaparin sodium 4000 UI twice daily and oxygen therapy 4 l/min, with a gradual reduction of dyspnea and clinical improvement. Given the absence of compressive and/or symptomatic complications related to lipomatous hypertrophy of the anterior RA free wall, surgical consultation was not required. Finally, on the ninth day from hospital admission, the patient was discharged with the indication to periodical pneumological visits and echocardiographic controls.

## Discussion

In the present case, a multi-instrumental evaluation, comprehensive of TTE implemented with PW-TDI, TEE, and CT angiography, allowed to quickly diagnose a severe PH secondary to ET as the principal cause of the patient’s dyspnea and a LAH of the RA free wall, as incidental finding without related symptoms. Interestingly, all examinations were performed during the patient's stay in the ED. CT angiography was preferred over cardiac magnetic resonance imaging (MRI), not available at our center.

It is important to consider LAH in the differential diagnosis of echocardiographic intracavitary RA masses. Different from endocardial vegetations, tumors, and thrombi, which have a pattern of incoherent motion, characterized by velocity and direction of movement independent and completely different from the surrounding myocardial and valve tissues, and without any correlation with the cardiac cycle [1-3]. In our case, the suspected RA mass observed during the TTE examination had a concordant motion with the anterior RA free wall and the antero-lateral tricuspid annulus and was in synchrony with the phases of the cardiac cycle. For these reasons, the suspected RA mass was reasonably considered a RA pseudomass, generated by the systolic RA free wall infolding into RA cavity. Both hypertrophy and hyperechogenicity of the anterior RA free wall were secondary to fatty infiltration. CT angiography confirmed the echocardiographic diagnosis of LAH.

As far as we know, to date, only two authors have reported LAH with RA free wall involvement [4,5]. Different from these authors, who described cases of deposition of adipose tissue both in the interatrial septum and in the RA free wall, in our case, the LAH was limited to a portion of the RA free wall only.

LAH is a histologically benign cardiac lesion characterized by excessive fat deposition (>2 cm fat infiltration) mostly in the region of the interatrial septum that spares the fossa ovalis with a pathognomonic dumbbell shape. Its prevalence ranges from 2.2% to 8% [6,7], depending on the diagnostic modality used for its detection (multislice CT vs. TEE, respectively). It may be associated with advanced age, female sex, and obesity [8]. Histologically, LAH is composed of mature fat cells. A great majority of patients with LAH are asymptomatic, and LAH is discovered incidentally at TTE [9]; however, in some cases, this fatty infiltration can either cause intra-atrial conduction disturbances and atrial arrhythmias, or in case of severe LAH, patients may develop obstruction of RA filling, shortness of breath, and/or heart failure symptoms [10,11]. The surgical treatment of LAH should be limited only to cases of symptomatic patients with life-threatening cardiovascular complications [12].

TTE is the preferred screening method for the diagnosis of LAH. It provides a detailed assessment of LAH location, size, and echogenicity. Due to increased spatial and temporal resolution, TEE may offer a better visualization of a small portion of LAH, as in the present case. Other imaging modalities, such as CT scan or MRI, may provide additional data, due to their high specificity in identifying fat. On CT scan, fat accumulation is detected as a homogenous hypodense mass with low density, which typically ranges from -65 to -120 Hounsfield units [13]. On MRI sequences, the complete signal loss of the mass on the fat suppression sequence is characteristic for the diagnosis of lipomatous hypertrophy [14,15].

In the present case, endomyocardial biopsy was not performed for confirming LAH diagnosis, due to the absence of cardiovascular complications related to LAH. Despite the lack of CT or MRI images with ECG gating, the comprehensive information derived from TTE, PW-TDI, TEE, and CT scan performed during the patient’s stay in the ED was considered compatible with LAH, rather than motion artifacts from pericardial fat.

## Conclusions

LAH with isolated RA free wall involvement is an extremely rare finding. It is a pseudomass that can mimic a cardiac tumor or a thrombotic mass. The implementation of TTE with PW-TDI may allow to quickly identify, among the intracavitary RA masses, those with features suggestive of benignity, such as the uniformly increased echogenicity and the concordant motion with the surrounding myocardial and valve tissues throughout the cardiac cycle, as in the present case. Its use should also be considered by echocardiographers in the ED.
